# PHGDH as a mechanism for resistance in metabolically-driven cancers

**DOI:** 10.20517/cdr.2020.46

**Published:** 2020-09-17

**Authors:** Richa Rathore, Charles R. Schutt, Brian A. Van Tine

**Affiliations:** ^1^Division of Medical Oncology, Washington University in St. Louis, St. Louis, MO 63110, USA.; ^2^Siteman Cancer Center, St. Louis, MO 63110, USA.

**Keywords:** PHGDH, cancer, metabolism, serine, one-carbon metabolism, folate cycle, drug resistance

## Abstract

At the forefront of cancer research is the rapidly evolving understanding of metabolic reprogramming within cancer cells. The expeditious adaptation to metabolic inhibition allows cells to evolve and acquire resistance to targeted treatments, which makes therapeutic exploitation complex but achievable. 3-phosphoglycerate dehydrogenase (PHGDH) is the rate-limiting enzyme of de novo serine biosynthesis and is highly expressed in a variety of cancers, including breast cancer, melanoma, and Ewing’s sarcoma. This review will investigate the role of PHGDH in normal biological processes, leading to the role of PHGDH in the progression of cancer. With an understanding of the molecular mechanisms by which PHGDH expression advances cancer growth, we will highlight the known mechanisms of resistance to cancer therapeutics facilitated by PHGDH biology and identify avenues for combatting PHGDH-driven resistance with inhibitors of PHGDH to allow for the development of effective metabolic therapies.

## Introduction

Recent advances in anti-cancer treatments have been based on the increased identification of biomarkers that allow for tumour-specific therapy. Biomarker-driven therapies allow for the differentiation between cancer and host cells, with the potential to decrease the side-effects often associated with chemotherapy in normal tissue. The hallmarks of cancer, such as rapid growth, sustained proliferation, and increased invasion and metastasis, can be traced to the activation or suppression of oncogenes, which can then be used as biomarkers for targeting therapeutics^[1]^.

The progression of cancer is dependent on the cellular metabolism of the tumour^[2]^. As such, developing therapeutic methods that target tumour metabolism has been a growing field. One-carbon metabolism is of importance in cancer metabolism, as this pathway is necessary for the *de novo* generation of biomass and other nutrient precursors. One-carbon metabolism consists of serine biosynthesis, betaine biosynthesis, the folate cycle, and the methionine cycle. The products of one-carbon metabolism contribute to nucleotide, lipid, and methylation metabolism, as well as nicotinamide adenine dinucleotide phosphate (NADPH), reactive oxygen species (ROS), and glutathione synthesis^[3,4]^. Of these, the serine biosynthetic pathway is of interest because the rate limiting enzyme, 3-phosphoglycerate dehydrogenase (PHGDH), is highly expressed in a variety of cancers and contributes to drug resistance. Both of these facets of PHGDH metabolism are discussed in detail below.

Importantly, tumour metabolism is highly adaptable, and the metabolic systems of cancer cells can reprogram in response to nutrient and anabolic precursor availability. As a result, there is a risk of innate or acquired drug resistance to metabolic inhibitors. In this review, the requirement of serine synthesis for cancer metabolism and tumour progression is explored. Through the biology of PHGDH, mechanisms of resistance to current cancer treatments, as well as proposed novel treatments, are identified. Understanding the mechanisms of resistance to metabolic treatments allows for the design of conditionally lethal combination therapies based on the inherent properties of tumour metabolism that can combat acquired resistance.

## The biological role of PHGDH in cancer

### PHGDH in the untransformed cell

**Figure 1:**
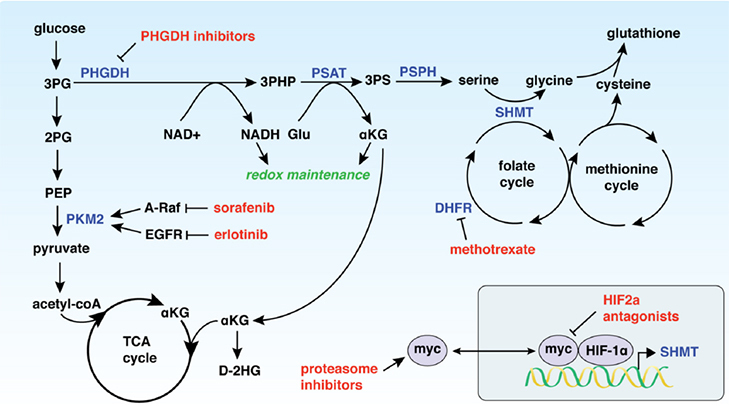
PHGDH expression drives resistance to a variety of chemotherapeutics (shown in red) through modulation of metabolic pathways. 3PG: 3-phosphoglycerate; 2PG: 2-phosphoglycerate; PEP: phosphoenolpyruvate; PKM2: pyruvate kinase M2; TCA cycle: citric acid cycle; αKG: α-ketoglutarate; D-2HG: D-2-hydroxyglutarate; PHGDH: 3-phosphoglycerate dehydrogenase; 3-PHP: 3-phosphohydroxypyruvate; PSAT: phosphoserine aminotransferase; 3PS: 3-phosphoserine; PSPH: phosphoserine phosphatase; NAD^+^: nicotinamide adenine dinucleotide, oxidized; NADH: nicotinamide adenine dinucleotide, reduced; Glu: glutamate; SHMT: serine hydroxymethyltransferase; DHFR: dihydrofolate reductase

PHGDH is the rate-limiting enzyme of *de novo* serine biosynthesis. PHGDH catalyses the conversion of the glycolytic intermediate 3-phosphoglycerate (3PG) to 3-phosphohydroxypyruvate (3PHP). The PHGDH enzymatic reaction utilizes nicotinamide adenine dinucleotide (oxidized form, NAD^+^; reduced form, NADH) as a cofactor, generating NADH as 3PHP is biosynthesized. Phosphoserine aminotransferase (PSAT1) subsequently uses glutamate to confer a nitrogen unit onto 3PHP, producing alpha-ketoglutarate (αKG) in addition to the serine precursor, 3-phosphoserine (3PS)^[5]^. Finally, phosphoserine phosphatase converts 3PS to serine [Figure 1]^[6]^.

**Figure 2:**
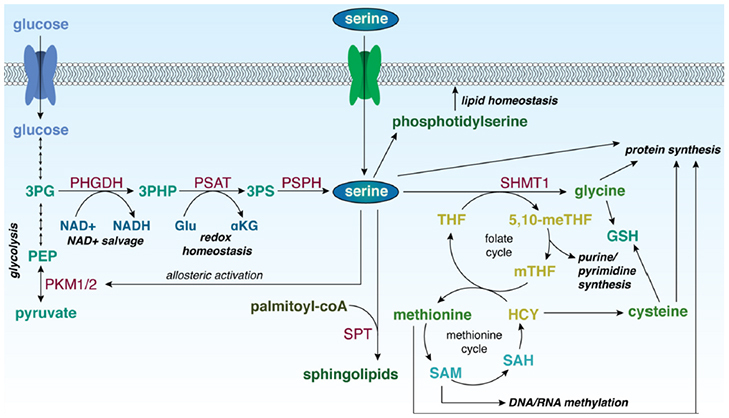
Role of serine in biological processes in the cell. 3PG: 3-phosphoglycerate; PEP: phosphoenolpyruvate; PKM1/2: pyruvate kinase isoforms M1/M2; PHGDH: 3-phosphoglycerate dehydrogenase; 3-PHP: 3-phosphohydroxypyruvate; PSAT: phosphoserine aminotransferase; 3PS: 3-phosphoserine; PSPH: phosphoserine phosphatase; NAD^+^: nicotinamide adenine dinucleotide, oxidized; NADH: nicotinamide adenine dinucleotide, reduced; Glu: glutamate; αKG: α-ketoglutarate; SPT: serine palmitoyltransferase; SHMT1: serine hydroxymethyltransferase 1; 5,10-meTHF: 5,10-methylene tetrahydrofolate; mTHF: 5-methyl tetrahydrofolate; THF: tetrahydrofolate; GSH: reduced glutathione; HCY: homocysteine; SAH: S-adenosyl homocysteine; SAM: S-adenosyl methionine

Serine is required for a variety of biosynthetic and signalling processes [Figure 2]. Serine itself can be used for protein and lipid biosynthesis^[7]^. The removal of a methylene unit from serine by serine hydroxymethyltransferase (cytosolic, SHMT1; mitochondrial, SHMT2) results in the synthesis of other amino acids, including glycine and, through intermediates of the methionine cycle, cysteine^[8,9]^. The methylene unit from serine also serves as a one-carbon donor for the folate cycle. The products of the folate cycle and the methionine cycle contribute to purine and pyrimidine synthesis, homocysteine recycling for DNA methylation processes, and the generation of NADH, NADPH, and adenosine triphosphate (ATP)^[10,11]^. Additionally, *de novo* serine biosynthesis utilizes glutamate and produces αKG, which can be converted to D-2-hydroxyglutarate (D-2HG), an oncometabolite^[5]^. As a result, the increased expression of PHGDH, as well as the other enzymes in the serine biosynthetic pathway, indicates that cells are utilizing these processes for proliferation and production of biomass.

Serine also plays a role in downstream signalling in the cell. When activated, pyruvate kinase (PK) catalyses the conversion of phosphoenolpyruvate to pyruvate, and is a key checkpoint for glycolysis^[12]^. As such, PK serves an important role in the occurrence of the Warburg effect, that describes tumour cells as more highly glycolytic than oxidative^[13]^. In its inactive form, PK shunts glucose carbons back through glycolytic intermediates, supporting the utilization of glucose for biomass synthesis rather than mitochondrial energy production^[14]^. PK exists in the body in two isoforms (isoform M1, PKM1; isoform M2, PKM2). PKM1 is mainly found in skeletal muscle and brain cells. PKM2 is expressed at a significantly higher ratio in proliferating cells and is the more dominant isoform in cancer; however, PKM1 has been shown to be expressed in certain cancers and cancer-associated fibroblasts as well^[13,15]^. Importantly, PKM2 is enzymatically activated by the direct binding of serine to an allosteric site^[16]^. Inactive PKM2 can therefore respond to changes in serine availability and direct glycolytic intermediates through 3PG into the biosynthesis of serine, activating PKM2. PKM2 expression is also mediated by a series of pathways, including phosphatidylinositol-3-kinase (PI3K)/AKT/mammalian target of rapamycin (mTOR)^[14,17]^.

### Incidences of decreased PHGDH

Serine deficiency can be extremely detrimental to cells, resulting in limited cellular proliferation and cell cycle arrest^[18]^. The first report of PHGDH deficiency was described in 1996, where two brothers with decreased plasma concentrations of serine and glycine presented with a severe neurological syndrome^[19]^. This decrease in serine and glycine was associated with decreased PHGDH expression and activity in the brains of the patients. Importantly, subsequent studies found that, although alternate pathways of obtaining serine exist, PHGDH deficiency resulted in significantly lower plasma serine levels^[20]^. Later studies found that phenotypes of PHGDH deficiency can exist on a spectrum and identified Neu-Laxova syndrome as a more severe example of PHGDH deficiency. Neu-Laxova syndrome is an autosomal recessive disorder caused by mutations to *PHGDH* and subsequent loss of serine, and is characterized by neurological impairment, impaired fetal development, and skeletal anomalies^[20,21]^.

At the cellular level, PHGDH deficiency can result in loss of DNA methylation. As the methylene unit provided to the folate cycle by the conversion of serine to glycine can transfer into the methionine cycle, serine indirectly supports the recycling of homocysteine to methionine, as well as the generation of precursors for S-adenosylmethionine (SAM)^[22,23]^. SAM is a common methyl-group donor required for DNA methylation^[24]^. In acute serine-starvation conditions, SAM is no longer used to methylate DNA and RNA^[25]^. Importantly, metabolic remodelling in cells during acute serine starvation allow for the upregulation of serine biosynthesis and serine uptake, to compensate for this loss^[26]^.

*De novo* serine biosynthesis also provides the precursors for the generation of phosphatidylserine and sphingolipids; PHGDH deficiency can alter sphingolipid homeostasis by prompting the generation of deoxysphingolipids^[27,28]^. Sphingolipids are produced by the enzyme serine palmitoyltransferase (SPT) that incorporates serine into palmitoyl-coA to produce a precursor to sphingosine. With decreased environmental serine, SPT instead utilizes alanine as a cofactor, resulting in the generation of deoxysphingolipids^[27,29]^. Deoxysphingolipids cannot be incorporated into cellular membranes, and increased levels of deoxysphingolipids can result in mitochondrial dysfunction^[30]^.

The results of decreased serine and loss of PHGDH activity are generally not beneficial to cells, unless extracellular serine concentrations are sufficiently high to support cellular utilization of the amino acid. The mechanism of negative regulation of PHGDH, therefore, is critical to explore. In melanoma, *PHGDH* expression is transcriptionally downregulated by wildtype p53^[31]^. It was found that *PHGDH* was a transcriptional target of the tumour suppressor p53, and that suppression of *PHGDH* resulted in promotion of apoptosis in p53-wildtype melanoma. These findings indicate the importance of PHGDH in the baseline functioning of the cell, but also highlight the utility of increased PHGDH as an oncogene in cancer.

## The tumorigenic consequences of elevated PHGDH

The increased expression of serine synthetic enzymes can signal that a cell is proliferating and generating biomass at a rapid rate, a hallmark of cancer^[32]^. PHGDH has been demonstrated to be upregulated in a wide variety of biologically distinct cancers, including colorectal cancer^[33]^, gastric cancer^[34]^, breast cancer^[35,36]^, melanoma^[37]^, Ewing’s sarcoma^[38]^, cervical cancer^[39]^, pancreatic cancer^[40]^, thyroid cancer^[41]^, colon cancer^[42]^, lung adenocarcinoma^[43]^, and non-small cell lung cancer^[44]^. Furthermore, increased PHGDH expression has been linked to brain metastasis^[45]^.

### Mutations to TP53

As previously stated, wildtype p53 can transcriptionally decrease gene expression of *PHGDH*^[31]^. Most of the cancers that present an overexpression of PHGDH harbour *TP53* mutations, including colorectal cancer (55%-60% *TP53* mutation), intestinal gastric cancer (66% *TP53* mutation), melanoma (85% *TP53* mutation), and non-small cell lung cancer (50% *TP53* mutation)^[46-49]^. This suggests that cancers with non-wildtype *TP53* may have increased PHGDH expression, though this has not yet been explored. Additionally, mutant p53 can regulate PKM2 through an mTOR-mediated phosphorylation at Tyr105^[50,51]^. The effects that mutant p53 can have on serine synthetic enzymes and downstream metabolic enzymes can contribute to mutant p53-driven tumorigenesis.

### Cell growth and proliferation

Increased PHGDH activity results in increased *de novo* serine biosynthesis. As previously described, serine supports a variety of cellular processes, including amino acid, nucleotide, and lipid synthesis, increased DNA methylation, and indirect αKG generation. *De novo* serine biosynthesis can in turn drive the synthesis of glycine, as well as the synthesis of cysteine from homocysteine within the methionine cycle, supporting protein synthesis^[8,52]^. Glycine is also directly incorporated into purine nucleotides. Furthermore, serine can be incorporated into lipids to produce phosphoserine and is a precursor to sphingosine, from which all sphingolipids are derived^[53]^. These processes support the generation of biomass and nucleic acid replication for the rapid proliferation of cancer cells.

### Redox homeostasis

The increased production of glycine and cysteine from serine can also contribute to maintenance of redox balance in cells, as these are the precursors for glutathione^[54]^. Glutathione, which exists in a reduced form (GSH) and an oxidized form (GSSG is the primary ROS scavenger of the cell^[55]^. Generation of αKG, an essential component of the citric acid (TCA) cycle, also contributes to maintaining redox balance, as αKG has antioxidative functions in the cell^[56]^. Furthermore, PHGDH can directly catalyse the conversion of αKG to D-2HG, an oncometabolite^[5]^. This reverse enzymatic activity requires the oxidation of NADH to NAD^+^, an important co-factor in metabolism and redox homeostasis^[5,57]^.

### The NAD^+^ salvage pathway

PHGDH utilizes NAD^+^ as a co-factor for enzymatic activity, producing NADH during the synthesis of 3PHP from 3PG^[58]^. In order to be utilized, NAD^+^ must be continually synthesized from tryptophan or regenerated from NADH. The NAD^+^ salvage pathway occurs through the recycling of nicotinamide to nicotinamide mononucleotide, and is therefore required for functional serine biosynthesis^[59]^. Conversely, mitochondrial serine catabolism has been demonstrated to supplement NADH levels through the folate cycle, suggesting that PHGDH and serine metabolism are directly regulated by NAD^+^/NADH availability^[60]^.

### Metastasis

Increased PHGDH expression has been demonstrated to not only promote cancer growth and proliferation, but also drive secondary tumour formation and metastasis^[61]^. In a study on lung metastasis, increased PHGDH increased hypoxia-inducible factor (HIF)-target gene expression. As increased PHGDH results in elevated production of glutathione, the resultant hypoxic conditions could be maintained by glutathione, subsequently executing metastatic programs^[61]^. Furthermore, in a study in brain metastases, increased PHGDH expression was correlated with increased metastatic potential to the brain. Interestingly, in this study, inhibiting PHGDH attenuated metastasis without affecting extra-cranial tumour growth, suggesting that the consequences of increased PHGDH expression were directly related to upregulated metastasis^[45]^. Finally, this study highlighted the limited environmental availability of serine in the brain, demonstrating the subsequent reliance on *de novo* serine biosynthesis^[45]^.

Taken together, baseline PHGDH expression contributes to *de novo* serine biosynthesis in the cell and supports a multitude of cellular pathways. Overexpression of PHGDH drives numerous pathways that are particularly useful for the initiation and progression of cancer.

## The role of increased PHGDH in cancer drug resistance

Given that increased PHGDH contributes to tumorigenesis, the role of PHGDH in cancer resistance is multi-faceted. Elevated PHGDH expression drives a reliance on certain metabolic pathways that cancer therapeutics directly target, thus resulting in a series of inhibitors to which cancers with increased PHGDH can develop resistance [Figure 1].

### Tyrosine kinases

Tyrosine kinases catalyse the phosphorylation of tyrosine residues, and have been shown to be constitutively active in oncogenic programs^[62]^. Sorafenib targets multiple tyrosine kinases, primarily the rapidly accelerated fibrosarcoma kinase (RAF) pathway, but also the vascular endothelial growth factor receptor and platelet-derived growth factor receptor pathways^[63,64]^. The inhibition of RAF-1 by sorafenib leads to inhibition of cellular proliferation and tumour growth. RAF-1 inhibition elevates ROS levels through stimulation of the Raf/MEK/Erk pathway, causing apoptosis^[65]^. Sorafenib has been approved for use in hepatocellular carcinoma (HCC), renal cell cancer, and thyroid cancer^[66]^. A study exploring the mechanisms driving sorafenib resistance found that increased PHGDH expression was a critical for this process in HCC^[64]^. As PHGDH and the serine synthesis pathway generate antioxidants (including glutathione and αKG), elevated PHGDH can combat the increased ROS levels induced by sorafenib treatment, thereby repressing apoptosis. Additionally, A-RAF, a RAF paralog, increases activation of PKM2 in the presence of serine, offering an additional target for sorafenib and another resistance mechanism in PHGDH-overexpressed cancers^[14,67]^.

### Epidermal growth factor receptor

Erlotinib is an inhibitor of epidermal growth factor receptor (EGFR), another tyrosine kinase associated with a number of signalling cascade pathways, including the Ras/Raf/MEF/ERK, PI3K/Akt, and STAT pathways^[68]^. The MEF/ERK pathway in particular links EGFR signalling to increased glycolysis, and Akt pathway signalling links EGFR to increased PKM2 activity^[69,70]^. EGFR has been demonstrated to be a driver of lung adenocarcinoma, and erlotinib treatment has been approved for treatment of non-small cell lung cancer in patients with and without EGFR mutations^[71,72]^.

Erlotinib binds to EGFR and inhibits downstream signalling cascades. Disruption of these signalling cascades results in decreased cell cycle progression, oxidative stress, and apoptosis. PHGDH is upregulated in erlotinib-resistant lung adenocarcinomas, likely due to the upregulation of glutathione and αKG synthesis as a cellular response to oxidative stress^[73]^. Furthermore, increased PHGDH expression and serine biosynthesis drives increased PKM2 activity and glycolysis, increasing the utilization of EGFR-related pathways.

### HIFs

HIFs contain two subunits that have transcription factor activity in hypoxic cells. The α-subunit HIF2α mediates redox homeostasis and can therefore modulate the effects of drugs such as sorafenib by decreasing resultant ROS levels and improving oxygen supply^[74]^. HIF2α is regulated by c-Myc activation and promotes hypoxic cell proliferation^[75]^. Therapies targeting HIF2α have therefore been explored as replacements for some tyrosine kinase inhibitors, such as sunitinib, in clear-cell renal cell carcinoma^[76]^. PHGDH is significantly overexpressed in HIF2α knockout tumours, as well as tumours that have shown sunitinib resistance^[76]^. The redox homeostasis maintenance conferred by PHGDH overexpression may be implicated here.

Interestingly, HIF1α, but not HIF2α, can also regulate the expression of SHMT, the enzyme that converts serine to glycine^[77]^. Increased SHMT1/2 expression can drive serine catabolism, increasing mitochondrial NADH production and fuelling the NAD^+^ salvage pathway required for PHGDH activity and serine biosynthesis^[59,77]^.

### The proteasome

Based on the potential role of c-Myc in resistance to HIF2α inhibitors, the role of NMYC in systems associated with PHGDH has been explored. NMYC activates ATF4, subsequently increasing PHGDH expression and activating a dependence on the serine biosynthetic pathway^[78-80]^. Proteasome inhibitors downregulate c-Myc, and have therefore been utilized to combat c-Myc-driven cancers^[81]^. Bortezomib is a proteasome inhibitor that has been highly effective for the treatment of multiple myeloma, a cancer in which c-Myc is highly active^[82]^. Proteasome inhibition by bortezomib results in the accumulation of unfolded proteins in the endoplasmic reticulum, resulting in cell death from the overproduction of reactive oxygen species (ROS)^[83]^. PHGDH is upregulated in bortezomib-resistant multiple myeloma; interestingly, this mechanism has also been identified as being through increased glutathione synthesis^[84-86]^ and subsequent ROS scavenging.

### Mitogen-activated protein kinase kinase

Mitogen-activated protein kinases (MAPK) and extracellular signal-regulated kinases (ERK) make up a series of proteins that transduce signals from the extracellular environment to inform cellular processes. This pathway can be overactive in some cancers, and activates transcription factors that are responsible for the progression of cancers such as melanoma^[87,88]^. In melanoma, tumour growth is enhanced through activation of the MAPK pathway, which is primarily driven by activating mutations in two oncogenes: *BRAF* and *NRAS*^[89]^. Mitogen-activated protein kinase kinase (MEK) enzymes are a part of the MAPK/ERK pathway, and activate the final kinases in this signalling pathway^[88]^. MEK inhibitors target this pathway and inhibit cell proliferation, ultimately causing apoptosis^[90]^. Importantly, *BRAF* mutations can increase susceptibility to MEK inhibitors; however, *NRAS* mutations that can over-activate MEK/ERK signalling can lead to resistance to MEK inhibitors^[87,91]^.

In an NRAS mutation model of melanoma, PHGDH was found to be upregulated. PHGDH is upregulated in melanoma at baseline, and is also upregulated in MEK inhibitor-resistant melanomas^[91,92]^. Overactive MEK/ERK signalling in BRAF- and NRAS-mutated cancers can overexpress PHGDH through ATF4 activation, driving utilization of the serine synthesis pathway to generate glutathione as a resistance mechanism. In addition, increased levels of folic acid, which can occur through increased flux of carbons from serine synthesis through the folate cycle, is a possible mechanism of resistance to MEK inhibitors in BRAF inhibitor-resistant melanomas^[91,92]^.

### Cisplatin

PHGDH is elevated in cervical adenocarcinoma, and is associated with poorer prognosis^[93]^. The first-line therapy for cervical adenocarcinoma is platinum-based chemotherapy. Cisplatin induces the DNA damage response in cells and causes mitochondrial apoptosis through Bcl2^[93]^. Given that PHGDH drives the indirect synthesis of nucleotides, upregulation of PHGDH could improve the DNA damage response in cells treated with cisplatin. PHGDH knockdown in cervical adenocarcinoma resulted in a decrease in Bcl2 expression, suggesting that baseline high PHGDH could also result in increased Bcl2, thereby mitigating the mitochondrial apoptotic response^[93]^. Interestingly, in ovarian carcinoma, increased PHGDH is a marker of cisplatin sensitivity, rather than resistance^[94]^. Further exploration of this and other pathways involved in cisplatin resistance is therefore needed.

### Other therapies

Elevated PHGDH also has the potential to play a role in resistance to therapies that have not yet been mechanistically explored. A critical therapy to therefore mention is the use of folate cycle inhibitors such as methotrexate and raltitrexed^[95]^. Methotrexate targets dihydrofolate reductase (DHFR), while raltitrexed targets thymidylate synthase (TYMS). These drugs require functioning folate cycles in order to be effective, and increased expression of folate-related enzymes is highlighted as a current mechanism of resistance^[96,97]^. As increased serine synthetic pathway activity can contribute more methyl units to the folate cycle, elevated PHGDH could be directly related to resistance to methotrexate treatment.

## The inhibition of PHGDH to combat chemo-resistance

### Single-agent inhibition of PHGDH

Given the increased expression of PHGDH in a variety of cancers, the single-agent inhibition of PHGDH seems to be a promising prospect for cancer therapy. A series of small molecule inhibitors against PHGDH have been developed, primarily targeting the enzymatic activity of PHGDH. These inhibitors include NCT-503, CBR-5884, PKUMDL-WQ-2101, BI-4924, and others under preclinical and clinical development^[35,98-102]^. It is important for the field to validate any finding with small-molecule inhibitors with knockdown and rescue or structural analysis to ensure that the effects of the drug are as a result of PHGDH biology and not an off-target effect of small-molecule inhibitors as a class of inhibitors. Iterations of these compounds have shown increasingly less off-target effects, with structure-based approaches used to synthesize PKUMDL-WQ-2101 in order to confirm specific binding to PHGDH^[101]^. PKUMDL-WQ-2101 and NCT-503 have been widely used in *in vitro* and *in vivo* research to interrogate the role of PHGDH and serine metabolism in normal and cancer cells^[101,103]^.

When cancer cells with elevated PHGDH expression are treated with high doses of NCT-503, cellular proliferation is attenuated and, in some cases, cell death is observed^[38,85,103]^. This phenotype can also be observed *in vivo*, as numerous studies have demonstrated that NCT-503 treatment results in decreased tumour growth for tumours and cell line-derived xenografts of PHGDH-high cancers. Knockout studies of PHGDH have also suggested mild suppressive effects on proliferation^[64]^. Furthermore, metabolites downstream of the serine biosynthetic pathway, such as one carbon units, folate intermediates, and pyrimidine intermediates, were also dysregulated by PHGDH inhibition^[102,103]^.

As of yet, direct PHGDH inhibition has not been tested in human clinical studies. However, as cells with increased PHGDH expression can develop resistance to apoptotic cell death by various drug treatments, cells treated with PHGDH inhibitors can rapidly alter their metabolism to take advantage of other mechanisms of fuel oxidation and redox maintenance^[104]^. As a result, PHGDH inhibition must be approached in a different way to optimize it for clinical development.

## Dual-agent inhibition of PHGDH and associated pathways

Given the highly adaptable nature of cancer metabolism, synergistic drug combinations are the future of metabolism-based drug resistance. Identifying increased PHGDH expression as a resistance mechanism for a variety of cancer therapeutics offers the opportunity to combine PHGDH inhibition with small molecule therapeutics. For example, increased PHGDH expression has been associated with both erlotinib and cisplatin resistance. Treatment with NCT-503 in these systems conferred sensitivity to the targeted therapy erlotinib and the chemotherapy cisplatin, respectively^[73,93]^. Furthermore, increased PHGDH expression was associated with resistance to sorafenib^[64]^. Treatment with NCT-503 mildly suppressed proliferation in hepatocellular carcinoma cells, but combining NCT-503 with sorafenib caused complete attenuation of proliferation and induced significant apoptosis^[64]^.

Beyond the known therapies that PHGDH confers resistance to, the combination of NCT-503 with the targeting of other cellular pathways can mitigate resistance. An understanding of the downstream mechanistic actions of PHGDH activity can unveil new therapies that could have action in PHGDH-overexpressed cancers. Given that PHGDH activity requires the NAD^+^ salvage pathway, a study that explored the use of NCT-503 with a nicotinamide phosphoribosyltransferase (NAMPT) inhibitor. This combination with a NAMPT that blocks the NAD^+^ salvage pathway resulted in synergistic cell death^[38]^. Recent studies have also explored the metabolic implications and pro-survival adaptations that occur as a result of PHGDH inhibition, implicating decreased TCA cycle activity, mTOR-independent and -dependent autophagy, and enhanced lipid metabolism and formation of lipid bodies^[28,103,105,106]^. The combination of PHGDH inhibition with inhibitors of these pro-survival metabolic adaptations should therefore yield synergistic and dramatic results in PHGDH-high cancers.

## Concluding remarks

3-phosphoglycerate dehydrogenase (PHGDH) expression in cancer has been linked to shorter progression-free survival, increased rates of metastasis, and poorer overall survival. An in-depth analysis of the biological consequences of enhanced PHGDH expression shows the links between *de novo* serine biosynthesis and a series of metabolic pathways that are targeted by current chemotherapies. In particular, cancers are capable of developing resistance to chemotherapies that induce apoptosis through increased ROS by increasing PHGDH, as PHGDH generates the necessary metabolic precursors for antioxidant and ROS scavenging activity. Therefore, increased levels of PHGDH, while contributing to tumorigenicity, can contribute to the innate or acquired resistance of cancers to current chemotherapies.

The direct inhibition of PHGDH by small-molecule inhibitors results in a decrease in cellular proliferation *in vitro*, with marginal inhibition of tumour growth *in vivo*. Inhibition of PHGDH also results in a series of metabolic adaptations that can acutely sensitize tumour cells to various chemotherapies. Current and future research on the adaptive mechanisms of resistance to PHGDH is needed to harness the upregulation of PHGDH in cancer. A multi-agent metabolic therapy can then be developed utilizing PHGDH as a biomarker for treatment efficacy and potential resistance.

## Declarations

### Copyright

© The Author(s) 2020.
